# The Division of In-Patient and Out-Patient Expenditure: King Edward's Hospital Fund for London Statistics


**Published:** 1915-03-20

**Authors:** 


### THE DIVISION OF IN-PATIENT AND OUT-PATIENT EXPENDITURE.

### King Edward's Hospital Fund for London Statistics.*

There are in London two special hospitals doing precisely the same class of work, each treating between 31,000 and 32,000 attendances of out-patients annually. The estimated cost at one is 1s. per attendance, and at the other, 1s. 8d. The result is that the hospital with the higher rate is in the position of deducting £1,000 more from its gross expenditure than the other before proceeding to calculate the cost per occupied bed. We will, for critical purposes, assume that the true cost in each case is an average between the two estimates. This would be 1s. 4d., and employing this flat rate in the cases of the two selected hospitals, we find the following striking difference between the cost per occupied bed, as shown in the King's Fund returns, and the cost after the average rate for out-patients has been applied.

*Hospital A*: King's Fund figures, £61 0s. 10d.; flat rate, £57 16s. 8d.

*Hospital B*: King's Fund figures, £68 8s. 1d.; flat rate, £70 7s.

As has been shown in previous articles, when the cost per occupied bed in different hospitals is under consideration it is necessary to give attention to a large number of influential factors, such as geographical position, whether there is a medical school, number of beds, the size of the staff, age of the charity and the building (involving questions of tradition and conditions which are difficult to alter), and other matters readily occurring to the student of hospital economics. Few of these conditions have influence to the same extent upon the cost of the out-patient department.

### Comparing Cost of Out-Patients.

When we come to compare the cost of out-patient attendances, especially where two hospitals doing identical work are concerned, it is difficult to suggest reasons why in one case it should be so much larger than in another, especially when we are aware that no part of the heaviest of the standing charges such as rent, rates, annual cleaning, renewals of furniture, and official salaries and pensions are included in the gross sum divided to find the unit.

Of the eightpenny excess in the attendance at Hospital B, three-halfpence is attributable to expenditure under the heading "Provisions," a rather smaller amount under "Surgery and Dispensary," about three-farthings under "Domestic," and nearly fivepence under "Salaries and Wages." (Hospital "A" under this heading claims only 6.15d., against 11.12d. of Hospital "B.") The difference is, therefore, caused by expenditure upon which the executive has a considerable amount of control from year to year. The ordinary observer will, therefore, find it difficult to solve the problem of why it should be twice as costly in the matter of staff to treat 30,000 out-patient attendances in one hospital as in another The officers and servants chiefly concerned would be the medical registrar, the staff of the $x$-ray department, the out-patient manager, out-patient porters, out-patient sister, and other members of the nursing staff concerned with the treatment of out-patients, doubtless a very small number compared with the in-patient nursing staff. We have here another perplexing omission of essential information, for the King's Fund return gives no indication as to what, if any, part of an almoner's or inquiry officer's department is included in the expenditure shown in the return. Such a department, if included, might account easily for twopence or threepence per attendance. In some hospitals, of course, there is a Samaritan Society which relieves the hospital of some of the cost of inquiry, and all work in connection with the after-care of patients. This may be, and probably is, an important matter in the cases of special hospitals such as those under review, and the unknown factor might well deserve the attention of those responsible for future issues of the report.

### Effect of Flat Rate on Cost per Bed.

We will now consider what would be the effect on the cost per bed of a "flat rate" for calculating the cost of out-patient attendances in the cases of sixteen larger general hospitals and six children's hospitals in London. The "flat rate" taken is

| Cost per bed under King's Fund system (lowest cost first). | £ | s. | d. | Cost per bed calculated on principle of a "flat rate" for out-patients (lowest cost first). | £ | s. | d. |
|---|---|---|---|---|---|---|---|
| **General Hospitals** | | | | ("*Flat Rate*," 8*d.*) | | | |
| 1. Seamen's ... | 58 | 6 | 4 | 1. Prince of Wales's ... | 53 | 0 | 10 |
| 2. Prince of Wales's ... | 62 | 2 | 7 | 2. Seamen's ... | 58 | 15 | 4 |
| 3. West London | 66 | 11 | 0 | 3. West London | 61 | 1 | 7 |
| 4. University College ... | 71 | 0 | 2 | 4. German ... | 72 | 13 | 5 |
| 5. German ... | 76 | 19 | 10 | 5. University College ... | 74 | 11 | 9 |
| 6. Middlesex ... | 78 | 4 | 11 | 6. Middlesex ... | 76 | 14 | 4 |
| 7. Great Northern Central ... | 80 | 10 | 1 | 7. Great Northern Central ... | 79 | 17 | 9 |
| 8. St. Mary's ... | 84 | 10 | 1 | 8. Guy's ... | 81 | 9 | 7 |
| 9. Metropolitan | 87 | 2 | 1 | 9. Metropolitan | 84 | 19 | 3 |
| 10. Guy's ... | 87 | 17 | 1 | 10. St. Mary's ... | 88 | 14 | 10 |
| 11. Charing Cross | 89 | 11 | 8 | 11. Charing Cross | 91 | 17 | 9 |
| 12. Westminster | 89 | 17 | 9 | 12. Westminster | 91 | 18 | 1 |
| 13. Royal Free | 92 | 10 | 11 | 13. Royal Free ... | 95 | 9 | 1 |
| 14. St. Thomas's | 92 | 15 | 3 | 14. St. Thomas's | 96 | 9 | 8 |
| 15. London ... | 93 | 6 | 1 | 15. St. George's | 97 | 11 | 4 |
| 16. St. George's | 96 | 6 | 4 | 16. London ... | 97 | 14 | 6 |
| **Hospitals for Children** | | | | ("*Flat Rate*," 7½*d.*) | | | |
| 1. Victoria ... | 63 | 4 | 9 | 1. Belgrave ... | 57 | 3 | 2 |
| 2. East London | 65 | 4 | 5 | 2. Victoria ... | 59 | 0 | 1 |
| 3. Paddington Green ... | 65 | 5 | 2 | 3. Paddington Green ... | 61 | 2 | 1 |
| 4. Great Ormond Street ... | 66 | 7 | 0 | 4. East London | 61 | 10 | 11 |
| 5. Belgrave ... | 68 | 8 | 6 | 5. Great Ormond Street ... | 70 | 15 | 0 |
| 6. Queen's ... | 72 | 5 | 11 | 6. Queen's ... | 72 | 19 | 6 |

* Previous articles appeared on October 10 and December 12, 1914, and January 23, February 6 and 20, 1915.

approximately the average based on the collective calculations of the hospitals concerned, and is the nearest halfpenny to the average of the rates employed in each group for the preparation of the figures in the first column.

While the children's list is altered completely, it will be noticed that the operation does not bring about a striking alteration of the order of the general hospitals in their rank as to lowness of cost, although in this particular there are some interesting changes of position; for instance, that of the Prince of Wales's, now in an apparently unassailable position at the head of the column. This general effect is, we think, fortuitous, as obviously, should the averages have been closer together, there would have been plenty of room for greater reverses of order with such remarkable decreases as £9 in the case of the Prince of Wales's, £5 the West London, and increases of £3 10s. at University College, £4 at St. Mary's, St. Thomas's, and the London.

Those interested in hospitals, but not working within them, are, as a rule, chiefly impressed by the cost per occupied bed; they do not understand that it is within the power of a hospital manager, with sufficient elasticity of conscience, to alter that figure materially by unburdening an over-generous amount of expenditure upon the gross amount to be divided up to make the cost per out-patient attendance. The discretion of the manager is practically uncontrolled and not readily questioned; he can easily justify his figures if called upon to do so, for no one knows better than he the peculiarities of his individual institution.

### The Secretary and the Business Manager.

The Supplementary Memorandum issued by the Hospital Funds in 1913 dismisses the difficulties of the process of dividing in-patient from out-patient expenditure with the remark, "Such apportionments, as is well known to all men of business, are regularly made in manufacturing concerns where a competitive business is done, and the cost of various goods manufactured on the premises has to be arrived at to enable contracts to be dealt with and the profit and loss thereon to be ascertained." But the man of business, when he fixes the price to the consumer, has always before him a knowledge of what other producers are charging. He knows he has to put on the market a golf ball at eighteenpence or a blend of tea at two shillings a pound, which will compare favourably in every respect with similar articles issuing from other houses. In a word, he is governed by the average price as fixed by the majority. He has to fix his rates of labour and buy his raw material with that end in view.

If there is anything in the analogy we have quoted and the deduction drawn from it, there is a good case for an assessment by a recognised authority of a rate to represent attendance cost.

There are, we suppose, very few institutions in this country where in-patient and out-patient expenditure can be calculated with anything approaching precision; we doubt if there is any instance where the expenditure can be precisely divided. Those hospitals able to carry through this operation in even a satisfactory manner are very few. In the case of the Hampstead General Hospital, which refers the whole of the out-patients to the old North-West London General Hospital (now amalgamated with the larger institution), and at Manchester where the Hospital for Sick Children at Pendlebury has an out-patient department nearer the centre of the city, some sort of satisfactory division can be made, but even in these cases there are some items, salaries and certain official expenditure under the heading of "Administration" and the like, where estimates must be made.

The task of division under the most favourable conditions is a troublesome one, and the more honestly the accountant carries it out, the more arduous is the work. The temptation to be on "the safe side," all the time knowing that the presentation of a respectably low cost per occupied bed is so desirable, is hard to withstand. If the computing authority had the solace of feeling that his labours were directed to some good and useful end, the labour would be cheerfully undertaken and carried through.

The assessment of a rate for each group of hospitals (dividing teaching institutions from the others in Group 1) would, we are sure, be accepted with alacrity by all concerned. The Central Funds have ample data for the preparation of such an assessment, and its use can be argued to be more scientific than the present haphazard and uncontrollable system.

